# Identification of Post-translationally Modified MHC Class I–Associated Peptides as Potential Cancer Immunotherapeutic Targets

**DOI:** 10.1016/j.mcpro.2025.100971

**Published:** 2025-04-14

**Authors:** Keira E. Mahoney, Larry Reser, Maria Virginia Ruiz Cuevas, Jennifer G. Abelin, Jeffrey Shabanowitz, Donald F. Hunt, Stacy A. Malaker

**Affiliations:** 1 Department of Chemistry, Yale University, New Haven, Connecticut, USA; 2 Department of Chemistry, University of Virginia, Charlottesville, Virginia, USA; 3 Proteomics Platform, Broad Institute of MIT and Harvard University, Cambridge, Massachusetts, USA

**Keywords:** MHC-associated peptides, immunopeptidomics, phosphorylation, post-translational modification, cancer immunotherapy

## Abstract

Over the past 3 decades, the Hunt laboratory has developed advancements in mass spectrometry–based technologies to enable the identification of peptides bound to major histocompatibility complex (MHC) molecules. The MHC class I processing pathway is responsible for presenting these peptides to circulating cytotoxic T cells, allowing them to recognize and eliminate malignant cells, many of which have aberrant signaling. Professor Hunt hypothesized that due to the dysregulation in phosphorylation in cancer that abnormal phosphopeptides could be presented by this pathway, and went on to demonstrate that this was, in fact, the case. Thereafter, the laboratory continued to sequence MHC-associated phosphopeptides and contributed several improved methods for their enrichment, detection, and sequencing. This article summarizes the most recent advancements in identification of modified MHC-associated peptides and includes the cumulative list of phosphopeptides sequenced by the Hunt lab. Further, many other post-translational modifications (PTMs) were found to modify MHC peptides, including O-GlcNAcylation, methylation, and kynurenine; in total, we present here a list of 2450 MHC-associated PTM peptides. Many of these were disease-specific and found across several patients, thus highlighting their potential as cancer immunotherapy targets. We are sharing this list with the field in hopes that it might be used in investigating this potential. Overall, the Hunt lab’s contributions have significantly advanced our understanding of antigen presentation and dysregulation of PTMs, supporting modern immunotherapy and vaccine development efforts.

## Highlights

• The Hunt lab has identified thousands of post-translationally modified MHC peptides.
• Detailed optimization procedures described improve MHC peptide sequencing.
• Bioinformatics analyses identified peptides for immunotherapy and vaccine design.

## Graphical Abstract

**Figure:**
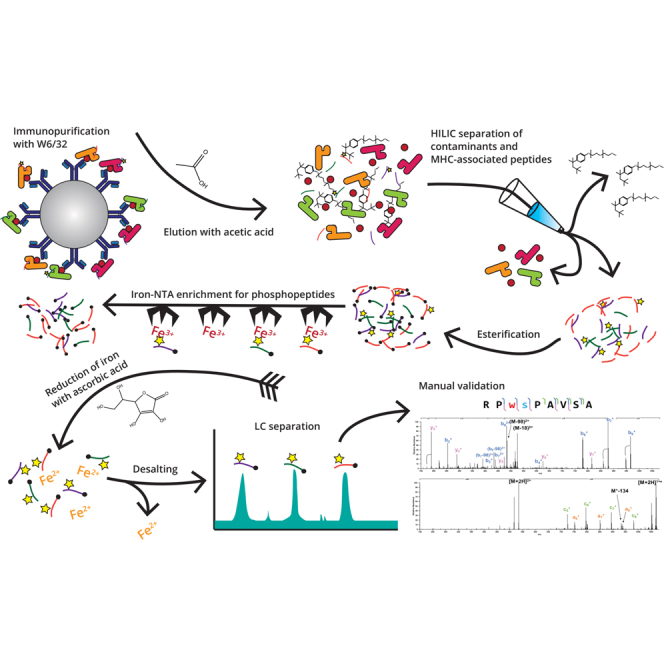

## In Brief

The Hunt laboratory spent nearly three decades advancing mass spectrometry–based technologies to identify peptides bound to major histocompatibility complex (MHC) molecules, crucial for presenting cellular health information to CD8+ T cells. This work has focused on MHC class I peptides with post-translational modifications (PTMs), which are often dysregulated in diseases like cancer. These findings underscore the potential of PTM peptides as targets for immunotherapy, and the lab’s contributions significantly advanced immunopeptidomics, with implications for cancer treatment and vaccine development.

**Fig. 1:**
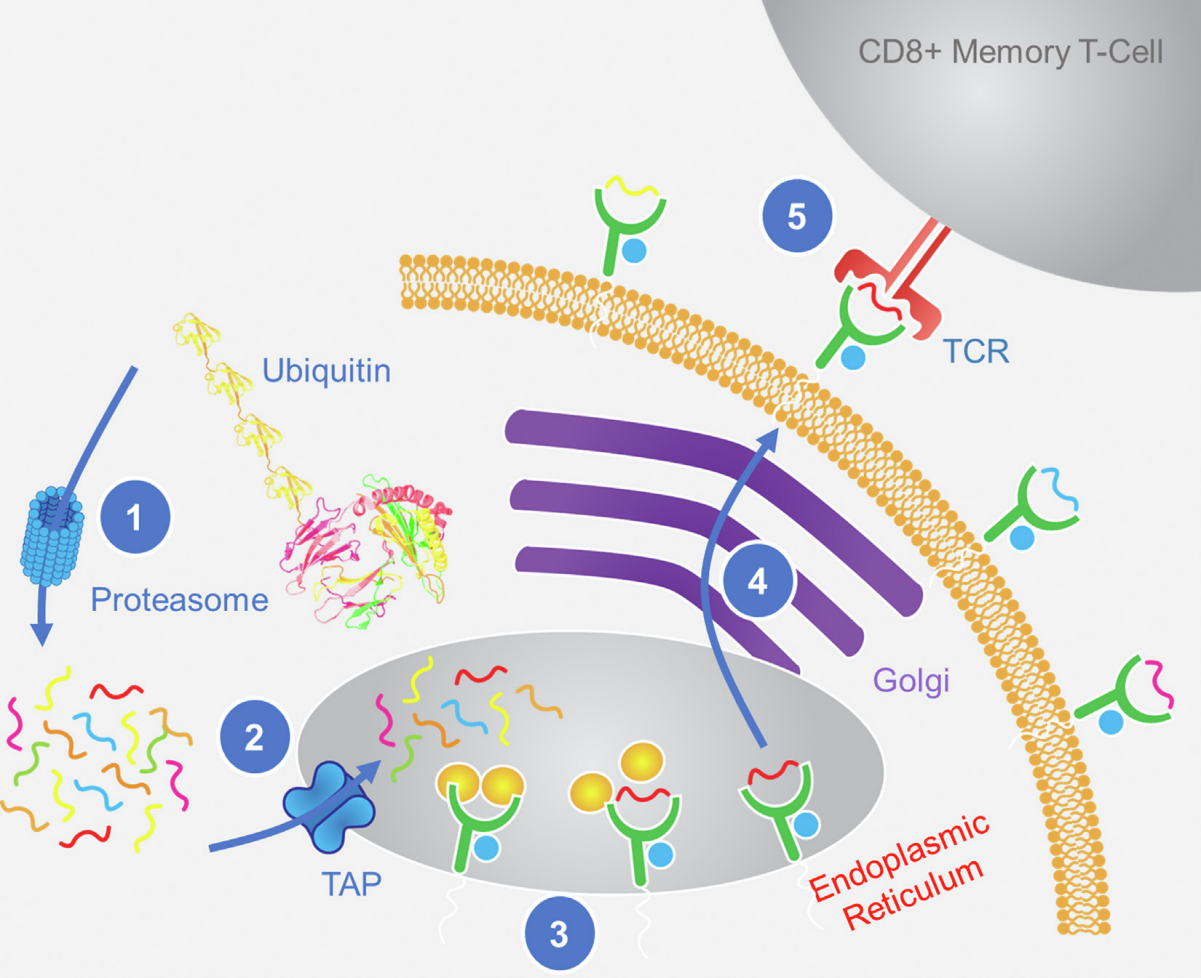
**The MHC class I processing pathway**. In all nucleated cells, cytosolic and nuclear proteins are degraded by proteasomes, transported to the ER to bind MHC molecules, and then presented on the cell surface for recognition by CD8+ T cells. Proteins are marked for degradation by ubiquitin, followed by digestion into peptides in the proteasome (1). Peptides are transported into the endoplasmic reticulum by TAP (2), where they associate with MHC molecules (3), and are then shuttled to the cell surface through the Golgi membrane (4). Finally, the peptide–MHC complex can be surveyed by CD8+ memory T cells for presence of infection or transformation (5). TAP, transporter associated with antigen processing.

The major histocompatibility complex (MHC) class I processing pathway is used by almost all nucleated cells as a way to display their health status to the immune system (Fig. 1) (1, 2, 3). To do this, cells present peptides derived from endogenous proteins, many of which were marked for degradation by ubiquitin and degraded by the proteasome. These peptides are then transferred to transporters associated with antigen processing into the endoplasmic reticulum, where the peptides are further shortened by peptidases, such as endoplasmic reticulum aminopeptidase 1 and 2, usually from 8 to 12 amino acid length peptides. Peptide binding stabilizes the MHC molecule, allowing for the MHC–peptide complex to be transported *via* the Golgi to the cell surface, where the peptides are displayed to circulating CD8+ T cells (1, 2, 3).

The human version of MHC class I is called the human leukocyte antigen (HLA) class I (4). The classic HLA alleles (HLA-A, HLA-B, and HLA-C) are all encoded by a highly polymorphic genomic region on chromosome 6. An individual can express up to six different allotypes (two each of A, B, C), as one of each type is inherited from each parent. Class I molecules are heterodimers consisting of a transmembrane heavy α-chain associated with a smaller β-2-microglobulin protein (β2m) (5). Variability between the different alleles occurs within the peptide binding groove of the α-chain, which results in diverse peptide binding motifs (6). The HLA molecule relies on interactions near the beginning and end of the peptide that anchor the peptide in the binding groove. At the beginning of the peptide, the second or third residue is the most common anchor site. The C-terminal anchor is usually the final residue of the peptide (7). Despite the limitations placed on peptide binding by the allelic motifs, each class I molecule can bind tens of thousands of different peptide sequences (5). Since each cell can present anywhere from thousands to hundreds of thousands of HLA molecules, each cell can display an extremely heterogeneous population of peptides on its surface. The expression level of each peptide on the cell surface can vary dramatically. Only a single copy of a peptide may be present, while others may have hundreds or thousands of copies on the cell surface. The center residues of the HLA-associated peptide are solvent exposed and capable of forming interactions with circulating CD8+ T cells.

Naïve CD8+ T cells are trained to differentiate antigenic peptides from healthy cellular expression through thymic education. Briefly, progenitor T cells, or thymocytes, are transported to the thymus (1, 3). Upon arrival in the thymus, they undergo a differentiation phase, wherein variable regions of genes encoding for T-cell receptor (TCR) proteins are rearranged to produce different binding affinities (6). After differentiation, they undergo a phase of intense proliferation. A period of positive selection follows, wherein only thymocytes that bind weakly to complexes of self-peptides and self-MHC are allowed to progress. This ensures that the receptors are able to recognize and associate with MHC complexes. During this process, the thymocytes will mature and begin to express higher levels of TCRs on the cell surface. At this point, thymocytes that bind too strongly to the self-peptide self-MHC complexes will be removed by negative selection, which prevents an autoimmune response to self-peptides. Approximately 98% of thymocytes are removed during thymic education, while the remaining 2 to 4% (about 1–2 million/day) are released to the peripheral immune system as naïve T cells (1). Naïve T cells then circulate through lymphoid organs and blood *via* the lymphatic system (8). Here, they are allowed to test their binding affinity with a variety of MHC-associated peptides. This primarily occurs in the lymph nodes with antigen-presenting cells (APCs). When the naïve CD8+ T cell associates strongly with an antigen presented on an APC, the APC releases costimulatory signals that will prompt its conversion to mature effector T cell and begin rapid proliferation (9). These effector T cells then begin to migrate through the body in search of antigen-positive cells. Upon encountering the antigen, cytotoxic T lymphocytes can release lytic vesicles containing perforin and granzyme into the synaptic region, causing lysis of the target cell (10).

Some diseases that are undetected by the immune system may generate minor changes in MHC peptide expression or be present in immune suppressive environments. To overcome these hurdles, the body can be primed to generate an immune response against a disease-specific HLA presented. Classes of immunotherapeutics that aim to harness the ability of T cells to identify and eliminate diseased cells include rationally designed T cell–targeted vaccines, T-cell therapies, and bispecific TCRs and antibodies that target HLA–peptide complexes (11, 12, 13, 14). Excitingly, varieties of these immunotherapeutics are in clinical trials and have had promising early results (15, 16, 17).

The field of immunopeptidomics has rapidly developed with much of the focus around the identification of MHC-associated peptides for cancer immunotherapeutics (18). Most of these efforts aim to identify peptides containing somatic mutations (neoantigens) that are exquisitely tumor specific (19, 20, 21). However, neoantigens may not be abundant is all cancer types and are often unique to each patient, which can limit their targeting in specific diseases (15, 22). Therefore, efforts continue to expand beyond neoantigens and identify HLA peptide antigens that are shared more broadly across different patients and tumor types for the development of future immunotherapies. One class of tumor-associated antigen that are being explored as potential shared cancer antigens are post-translationally modified (PTM) HLA peptides. This is because the ability of diseases, such as cancer, to successfully evade the immune system is dependent on dysregulated cell signaling, which is very tightly controlled in normal cells and relies on PTMs to mediate transcription and translation of cell cycle proteins (23, 24). In diseased cells, various PTMs are dysregulated, occurring more often and/or on amino acids they may not normally modify. Peptides derived from these aberrantly post-translationally modified proteins can be presented on the cell surface by MHC-I and can be recognized as abnormal by circulating CD8+ T cells. However, peptides with PTMs are estimated to be present at approximately 0.1 to 1% of the total HLA-associated peptide population, thus making them difficult to detect by conventional methods without enrichment (25, 26, 27, 28). Therefore, methods to enrich PTM-containing HLA peptides are necessary to improve our ability to detect and better understand the underlying biology of HLA-presented peptides.

The Hunt laboratory pioneered the field of mass spectrometry–based immunopeptidomics with the underlying goal to identify disease-associated PTM peptides for use in immunotherapeutics, as detailed in the perspective by Abelin and Cox (29). During these endeavors, the idea that disease-associated PTM HLA peptides may represent a class of antigens was corroborated by the fact that a subset of PTM peptides demonstrated an ability to stimulate healthy donor and patient T-cell responses (30, 31, 32, 33, 34). Together, this leads us to believe that disease-associated PTM peptides are putative candidates for T cell–targeting immunotherapy.

In this article, we present the Hunt Lab’s nearly 30-year effort to sequence HLA-associated PTM peptides. In particular, we highlight recent key methodological developments that allowed for the identification of over 2450 peptides, many of which were disease-specific and shared between patients. Ultimately, we hope to share the laboratory’s legacy and provide a useful resource for those interested in investigating neoantigens in cancer.

## Experimental Procedures

We note that the methods described here are those currently used by our laboratories; however, these techniques evolved dramatically over time. For historical discussion of developments in the fields of immunopeptidomics and mass spectrometry instrumentation, we point the reader to perspectives highlighted in this issue, especially the one written by Abelin and Cox (29).

### Experimental Design and Statistical Rationale

We are unable to give a formal response to the total number of samples analyzed as the study was performed over the course of 30 years. We estimate that our final list of peptides was generated from over 250 samples and hundreds, if not thousands, of raw files. In general, replicates were not performed in this study as we were often using tumor tissue from patients, and the entire tumor sample would be used for one experiment. Where possible, we compared tumor tissue to adjacent normal tissue from patients; this was not always available. Otherwise, to understand sample loss, phosphopeptide standards were added to samples prior to sample processing.

### Immunopurification of MHC-Associated Peptides

MHC-bound peptides were isolated as described previously, described here in brief (35). First, the pan-human class I antibody W6/32 was conjugated to NHS-Sepharose beads. For each gram of tissue (or 1E9 cells), 3 mg of antibody conjugated *via* reductive amination to 300 μl of beads were used. Beads were washed twice in PBS and incubated rotating overnight with antibody. The following day beads were pelleted, blocked for 1 h with 100 mM Tris–HCl, and then washed twice in alternating solutions of 100 mM ammonium acetate pH 5 with 500 mM NaCl and 100 mM Tris buffer pH 8. Beads were resuspended in 20 mM Tris, 150 mM NaCl up to 1 mg/ml remaining antibody, and stored at 4 °C until use.

Cells were lysed in a buffer of 20 mM Tris–HCl, 150 mM NaCl, 1% CHAPS, pH 8 supplemented with protease and phosphatase inhibitor cocktails I and II (Sigma-Aldrich). Buffer was added to the cells at 5 ml per 1e9 cells or 1 g of tissue. Frozen cells were resuspended in lysis buffer before rotating at 4 °C for 1 to 2 h. Lysed cells were ultracentrifuged at 10,000*g* for 1 h at 4 °C. The supernatant was then mixed with the antibody-bead conjugates and rotated at 4 °C overnight to allow MHC binding. The following day, pelleted beads were washed in 10 ml of lysis buffer, resuspended in ∼500 μl of 20 mM Tris–HCl, and transferred to a microcentrifuge tube or polypropylene column. Beads are then washed in a volume of 1 ml with: 2 × 20 mM Tris–HCL, 150 mM NaCl; 2 × 20 mM Tris–HCl, 1 M NaCl; 3 × 1 ml 20 mM Tris–HCl. Washed beads were then spun through a prewet 3 kDa Amicon Ultra centrifugal filter column and all liquid removed by centrifugation. Columns were covered with parafilm and stored at −80 °C for shipment.

### Sample Cleanup—C18

The sample was reconstituted in 100 μl of 20 mM Tris pH8 with 2 mM MgCl_2_. Benzonase nuclease (5 units) were added, and the sample was incubated at 37 °C for 1 h. Stop and go extraction (STAGE) tips were fabricated with two cores as previously described (82). Samples larger than 3E8 cell equivalents were processed on multiple tips to reduce clogging. STAGE tips were equilibrated using the following wash steps: two washes with 100 μl of methanol, one wash with 100 μl of 80% acetonitrile/0.1% acetic acid at 1500*g*, and two washes with 100 μl of 1% acetic acid at 3500*g*. Thawed beads were transferred from the filter to a low-protein binding tube using two transfers in 200 μl of water. After centrifugation, the supernatant was removed and set aside.

To elute the peptides from HLA molecules bound to beads, 150 μl of 10% acetic acid was added to the tube and placed in a shaker for 5 min at room temperature. The beads were centrifuged at 300*g* for 1 min and the supernatant transferred to a low-protein binding tube. This process was repeated to ensure complete elution of peptides from HLA. Two internal phosphopeptide standards were added to the 10% acetic acid elution to determine recovery from the cleanup process. The elution supernatant was loaded onto the STAGE tips in 150 μl aliquots, centrifuging at 3500*g* until the entire volume had passed through. STAGE tips were washed twice using 100 μl of 1% acetic acid. Peptides were eluted from the STAGE tips using a stepped acetonitrile gradient: 20 μl of 20% acetonitrile/0.1% acetic acid, 20 μl of 40% acetonitrile/0.1% acetic acid, and 20 μl of 60% acetonitrile/0.1% acetic acid. Eluted peptide fractions were dried to completion using a Centrivap, reconstituted in 0.1% acetic acid to a concentration of 1E7 cell equivalents per μl (or 1 mg/μl for tissue), and stored at −35 °C.

### Sample Cleanup—HILIC

Spin tips were fabricated by using a 16-gauge blunt tip needle to cut a core from a Whatman glass fiber filter and pressurizing the filter into a 200 μl pipette tip. Slurry was then made, using approximately 10 μl of polyhydroxyethyl aspartamide (PHEA) material (12 μm or 20 μm diameter, 200 Å pores) and 200 μl of 200 mM ammonium acetate (pH 6) per tip. The material was added and centrifuged at 350*g* for 1 min. The 10 μl of material created a packed bed length of approximately 5 mm over the glass fiber filter. The material was then rinsed using 100 μl of the following solvents and spun for 1 to 2 min at 300 to 400*g*: 0.5% formic acid, 90% acetonitrile 20 mM ammonium formate (pH 3), and 200 mM ammonium formate (pH 3), and water. The columns were then equilibrated twice using 90% acetonitrile with 20 mM ammonium formate. Dried samples were reconstituted in 10 μl of 200 mM ammonium formate before adding 90 μl of acetonitrile and using a vortex mixer. The reconstituted sample was then added. Then, 100 μl of 90% acetonitrile 20 mM ammonium formate was used to rinse the sample. Peptides were then eluted by adding 100 μl of 50% acetonitrile in 0.2% acetic acid to the column twice. Proteins were eluted for further analysis using 200 μl of 0.5% formic acid. After cleanup, samples were dried down and reconstituted in 0.25 μl glacial acetic acid and diluted with 15 μl of LC/MS grade water.

### Sample Screening

Before performing an enrichment, the unenriched sample was analyzed to verify successful immunoprecipitation and determine the level of peptidic content in the samples. A precolumn (PC) was attached to an HPLC and equilibrated with 100 mM acetic acid for 5 to 10 min at approximately 5 μl/min. One microliter of the reconstituted sample was added to 10 μl of 0.1% acetic acid and loaded by pressure vessel onto the PC at 1 μl/min. After loading, the sample was desalted on the HPLC for 5 to 10 min at approximately 5 μl/min. After desalting, 100 fmol of angiotensin I and vasoactive intestinal peptide was loaded *via* pressure vessel, rinsed for 30 s on the HPLC, and the column was reattached to the analytical column. The column was allowed to equilibrate for 15 min at approximately 40 bar (∼100 nl/min) before beginning data collection on the mass spectrometer. A 40-min gradient was run from 0 to 40% acetonitrile with 100 mM acetic acid. Full mass spectrometry (MS1) scans were collected at 60,000 resolution in the Orbitrap of an LTQ-Orbitrap or the ion cyclotron resonance cell of a Fourier-transform ion cyclotron resonance. Both collision-activated dissociation (CAD) and electron transfer dissociation (ETD) MS2 scans were acquired in the ion trap, fragmenting the four most abundant ions with charge states of +2 or +3. After two sets of MS2 scans were taken in 10 s, masses were excluded from selection for 10 s. CAD was performed for 30 ms using a max inject time of 100 ms. ETD parameters included: 35 to 45 ms reaction time (instrument-dependent), Fourier-transform MS automatic gain control target of 2e5 charges, ion trap MS automatic gain control target of 1e4, and ETD reagent target of 2e5.

If samples were being investigated for the presence of glycopeptides, the instrument method was a top-speed higher energy collisional dissociation (HCD) triggered ETD when three of six O-GlcNAc fingerprint ions (*m/z* 204, 186, 168, 144, 138, and 126) were detected at >5% relative abundance. Peptide sequences were determined by manual interpretation of HCD, CAD, and ETD mass spectra.

The collected data were searched against the human proteome using Byonic (Protein Metrics) with MS1 tolerance at 10 ppm and MS2 tolerance at 0.35 Da, allowing two variable modifications of oxidation (Met), phosphorylation (Ser, Thr, Tyr), or cysteinylation. The results were filtered to only include identifications with a score greater than 300 and a |logProb| greater than 3. Relative abundances of peptides were calculated by comparing the peptide peak area to the average peak areas of two internal standard peptides. The amount of material used for an enrichment was then decided based on a combination of the peptide content in the screen and the amount of sample available.

### Fischer-Speier Esterification

The selected amount of sample, based on the unenriched analysis, was moved to a new 1.5 ml microcentrifuge tube and dried with a vacuum concentrator. To remove any residual water, the sample was dried twice more after additions of 50 μl methanol. Then, 80 μl of 3 M anhydrous hydrogen chloride in methanol was added to the sample, vortexed, bath sonicated for 10 s, centrifuged, and allowed to react for 1 h. The process was then repeated after drying once with methanol.

### Phosphopeptide Enrichment

For the preparation of Fe-nitrilotriacetic acid (NTA) resin using a 10 kDa molecular weight cut-off filter, all forward spins were performed at 14,000*g* for 5 min and all spins with the filter inverted (reverse spins) at 1000*g* for 1 min. A 10 kDa spin filter was rinsed twice with 500 μl of 0.01% acetic acid using one forward and one reverse spin during each round. Nickel (Ni)-NTA resin was removed from a Qiagen Ni-NTA spin column, reconstituted in water (1 mg/ml), and added to the spin filter in three aliquots, each followed by a forward spin. When adding solutions to the spin filter, the mixture was aspirated to remove any resin that had accumulated on the rim of the filter. The resin was rinsed using the following steps: two rinses with 450 μl of water using two forward spins, two rinses with 450 μl of 50 mM EDTA using two forward spins, and two rinses with 450 μl of water using two forward spins. The resin was activated by three rounds of 450 μl of filtered 100 mM FeCl_3_ using three forward spins. The activated resin was washed using the following steps: one rinse with 450 μl of 0.01% acetic acid using one forward spin, two rinses of 450 μl of 15% acetonitrile in 0.01% acetic acid using two forward spins, and one rinse with 450 μl of 0.01% acetic acid using one forward spin. The prepared Fe-NTA resin was stored in 0.01% acetic acid at 4 °C for up to 1 month.

A fused silica microcapillary column (360 μm o.d. × 150 μm i.d.) equipped with a 2 mm Kasil frit was packed with 2.5 cm of previously prepared Fe-NTA resin. The column was reactivated by pressure loading 100 mM FeCl_3_ for 10 min at a flow rate of 20 μlmin^-1^ followed by a 3-min incubation period in which the iron was allowed to incubate on the column. This process was repeated two times to ensure complete activation.

The activated column was equilibrated with 25 μl of 0.01% acetic acid at a flow rate of 0.5 μlmin^-1^. The dried, esterified peptide sample was reconstituted in 50 μl of 1:1:1 (methanol: acetonitrile: 0.01% acetic acid (vol/vol)) and pressure loaded onto the activated immobilized metal affinity chromatography (IMAC) column at a flow rate of 0.5 μlmin^-1^. Following sample loading, 25 μl of 1:1:1 was added to the sample tube and loaded onto the IMAC column at a flow rate of 0.5 μlmin^-1^. A final rinse with 15 μl of 0.1% acetic acid at a flow rate of 0.5 μlmin^−1^ was completed. The sample flow-through, 1:1:1 rinse, and 0.1% acetic acid rinse were collected in an Eppendorf tube and stored at −35 °C.

At this point, a PC was rinsed on a HPLC with solvent A at 30 bar for 10 min. The PC was connected to the end of the IMAC column with a Teflon sleeve and the IMAC-PC column was rinsed with 0.01% acetic acid for 10 min at a flow rate of 0.5 μlmin^-1^ to ensure that no leaks were present. Phosphopeptides were eluted directly onto the PC by pressure loading 15 μl of fresh 250 mM L-ascorbic acid in water (pH 2) at a flow rate of 0.5 to 1 μlmin^-1^. The column was then rinsed with 5 μl of 0.01% acetic acid before disconnecting from the PC.

The PC was rinsed on the HPLC for 20 min at a pressure of 30 bar. Standard peptides (100 fmol) were then loaded on the PC and the column was rinsed for an additional 30 s on the HPLC before connecting to the analytical column (10–12 cm of 3 μm Dr Maisch Reprosil Pur AQ in 360 o.d. 75 i.d. fused silica). The connected columns were equilibrated on the HPLC for 15 min at 45 bar before recording began. Peptides were separated on a gradient from 0 to 40% MeCN in 100 mM acetic acid at 45 bar with a flow rate of ∼150 nlmin^−1^. Representative data files can be accessed *via* PRIDE with the accession number PXD061414.

### O-GlcNAc Enrichment Methods

For some of the O-GlcNAc peptides, enrichment *via* amino phenylboronic acid (APBA) was performed, as described previously (36). After the introduction of the Thermo Orbitrap Tribrid series, this enrichment was generally rendered unnecessary due to the speed and sensitivity of the instrument. For the enrichment, though, POROS20 beads (7 mg) were dispersed into 200 μl of PBS (pH 6–7) containing 40 μmol of APBA. Following the addition of NaCNBH_3_ (1.3 μmol in 1 μl of PBS), the reaction was allowed to proceed with agitation for 2 h at room temperature and then quenched by washing the beads with water on a spin column (pore size <20 μm). Water was removed under vacuum and the dried beads were stored at 4 °C.

Class I MHC peptides from 2 × 10^8^ to 5 × 10^8^ cells in 0.1% acetic acid were desalted by loading the solution onto a fused-silica column (360 μm o.d. × 150 μm i.d.) packed in-house with 5 cm of irregular C18 (5–20 μm diameter) particles at a flow rate of 0.5 μl/min. After washing the column with 25 μl of 0.1% acetic acid, peptides were eluted into Eppendorf tubes with a 40-min gradient (0–80%) solvent B (A: 0.1 mol/l acetic acid, B: 70% acetonitrile, 0.1 mol/l acetic acid). Fractions were screened by MS and those that contained peptides, but not CHAPS detergent, were combined, taken to dryness and stored at −35 °C.

APBA beads were washed 3× with 100 μl of anhydrous dimethyl formamide and then allowed to react with desalted peptides in 20 μl of anhydrous dimethyl formamide for 1 h with agitation at room temperature. Solvent was removed by centrifugation, and the beads were washed 2× with 100 μl of anhydrous acetonitrile. Bound peptides were released by agitating the beads in 20 μl of 0.1 mol/l acetic acid for 30 min. Supernatant was collected, taken to dryness, and reconstituted in 10 μl of 0.1 mol/l acetic acid for loading onto an in-house packed C18 column for MS analysis.

### Mass Spectrometric Methods

Analyses of THP1 and VMM39 were performed on an LTQ Orbitrap XL and LTQ FT Ultra, respectively, both equipped with front-end ETD sources. High-resolution MS1s were taken at 60,000 resolution with a scan range from 300 to 1500 *m/z*. Data were collected in a data-dependent manner, with the five most abundant ions selected for fragmentation with an isolation window of 3 Da. After being selected twice for fragmentation in 10 s, the masses were put on an exclusion list for 10 sec. Only peptides with a charge of two or three were selected. For each precursor selected for fragmentation, low-resolution MS2s were collected at a normal scan rate. A pair of MS2s was collected for each precursor: HCD fragmentation at 35% normalized collision energy (nCE) and ETD fragmentation with a reaction time of 50 ms.

Analyses of HeLa and JYA2 were performed using an Orbitrap Fusion Lumos Tribrid mass spectrometer. All other samples were analyzed using the Orbitrap Fusion Tribrid mass spectrometer. MS1 scans were collected in the Orbitrap at a resolution of 60,000 and a scan range of 300 to 1500 *m/z*. Data were collected in a data-dependent manner, with the precursors selected for fragmentation in order of decreasing intensity using a top speed method with a 3 s cycle. Precursors were isolated by quadrupole isolation with a window of 1.8 Da. Singly charged precursors were isolated with an offset of 0.4 Da to capture the C13 isotope peak. Based on charge state, precursors were subjected to any of three fragmentation types: (1) Fragmentation with CAD using 30% nCE collected at 7500 resolution with an inject target of 100,000 ions and maximum inject time of 120 ms, (2) fragmentation with HCD using 27% nCE but otherwise the same as CAD, and/or (3) fragmentation with ETD collected at a rapid scan rate in the ion trap with a target of 30,000 ions with 50 ms maximum inject time. Singly charged precursors were only selected if their precursor mass was greater than 850 *m/z* and they were subjected to HCD fragmentation. Doubly charged precursors between 350 and 950 *m/z* were subjected to CAD fragmentation and ETD fragmentation with 60 ms reaction time. Precursors with charge states of three or four were selected between 300 and 650 *m/z* and underwent HCD fragmentation as well as ETD fragmentation with a charge-calibrated reaction time.

### Data Analysis

Data files were searched using the Byonic (Protein Metrics) search algorithm against the human database (SwissProt with isoforms) and against an in-house database of previously identified phosphorylated MHC antigens. An MS1 tolerance of 10 ppm and an MS2 tolerance of 0.35 Da were allowed. In the case of high-resolution analysis, the MS2 tolerance was changed to 20 ppm. A fixed methylation was set on the C terminus, aspartate, and glutamate with one variable demethylation set as a rare modification. Two oxidized methionine and two phosphorylated serine, threonine, or tyrosine were allowed as common variable modifications. Homocysteinylated cysteine was allowed as a single rare modification. A maximum of one rare modification and three common modifications were allowed. The search algorithm identifications were then manually validated and verified by a second party. For positive identifications, significant sequence coverage, signal-to-noise ratio and accurate mass were required. In the case of modified peptides, a site was only considered localized when fragment ions could definitively confirm its location; for labile modifications, this was done primarily using ETD. In some cases (most prior to 2010), synthetic peptides were generated to confirm correct elution and fragmentation profiles. Representative annotated spectra have been uploaded to PRIDE with the accession number PXD061414.

### In-House MHC Antigen Database

Verified post-translationally modified MHC antigens were added to an in-house database (supplemental Tables S1–S4). Metadata recorded for each antigen included the type of modification, extent of site localization, UniProt ID, and sample IDs. Detailed information about sample origin, source, antibody used for purification, and HLA allele(s) can be found in supplemental Table S5. Abbreviations regarding cancer type can be found in supplemental Table S6.

### Bioinformatics Analysis

Phosphorylated MHC peptides were categorized into six groups: immortalized human cell lines, human cell line mouse xenograft models, nontumor patient tissue, tumor patient tissue, T cells, and cadaverous brain tissue. Localized phosphosites were cross-referenced to PhosphoSitePlus (37), UniProt PTM data (38), and the PRIDE human phosphoproteome map (39) to identify previously reported sites. Gene set enrichment of site-localized tumor phosphopeptides (929 peptides, 631 proteins) was done using ShinyGO (version 0.80, http://bioinformatics.sdstate.edu/go80/) (40) with a background gene list derived from tissue-matched MHC class I peptides (56,141 peptides, 11,028 proteins) identified by the HLA Ligand Atlas (41). Enriched gene ontology molecular function terms were estimated using an FDR cutoff of 0.01 and a minimum pathway size of 10.

BamQuery (42) analysis was performed using the unmodified base sequences of phosphorylated MHC antigens 8 to 12 amino acids in length. The background RNA-seq dataset included 2602 BAM files from 55 genotype-tissue expression tissues and 30 BAM files from medullary thymic epithelial cells and dendritic cells. Peptides were classified as tumor antigens if their average RNA expression was below 8.55 RPHM. Genomic origin was predicted using the highest RNA-seq read count among all genomic locations. Tumor antigens were cross-referenced with the Immune Epitope Database (43) to identify epitopes that have been previously studied for immune reactivity.

## Results

**Fig. 2:**
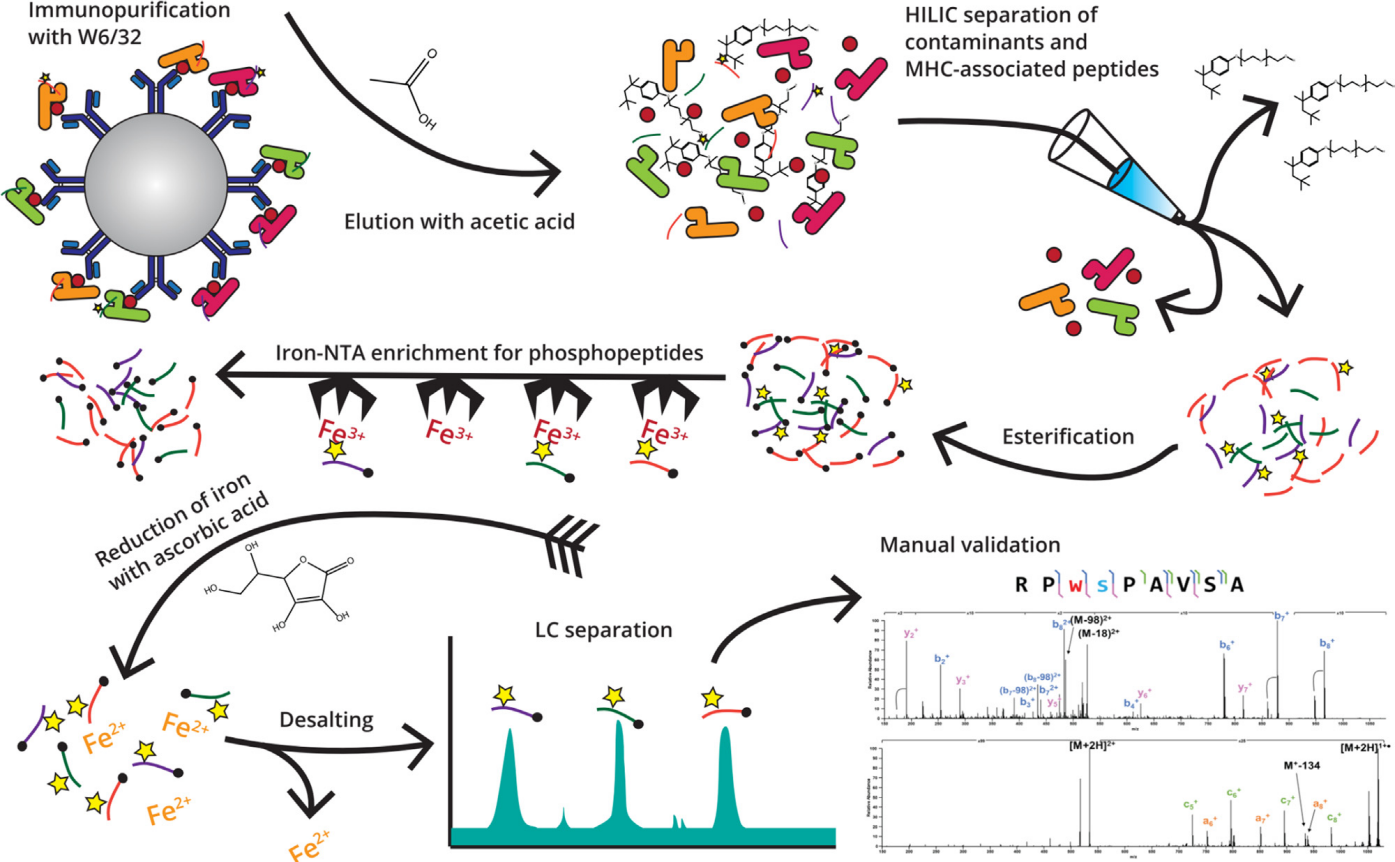
**Optimized MHC-associated phosphopeptide enrichment procedure**. Peptides are first immunopurified with a pan-class I antibody (W6/32) and eluted with acetic acid. Contaminants are removed using a HILIC-based cleanup, followed by Fischer esterification and iron-NTA enrichment of phosphopeptides. The iron is reduced with ascorbic acid which releases phosphopeptides, and then the resulting elution is desalted, separated by reversed-phase HPLC, and analyzed by mass spectrometry. All resulting spectra were manually validated. HILIC, hydrophilic interaction chromatography; NTA, nitrilotriacetic acid.

As discussed above, the Hunt laboratory has contributed significantly to the field of immunopeptidomics and mass spectrometry instrumentation development (29). While our previous protocols (i.e., pre-2015) allowed for identification of tens to hundreds of phosphopeptides with less starting material than is generally used in the field for unmodified peptide analysis, we recently identified several potential areas for improvement in nearly every aspect of our phosphopeptide enrichment procedure. By optimizing these methods, we can now regularly detect hundreds of phosphopeptides in a single experiment. Here, we highlight the optimization tools that allowed us to vastly enhance our total list of post-translationally modified HLA-associated peptides; a summary of the optimized workflow is shown in Figure 2.

### Application of PHEA Cleanup for Contaminant Removal

When MHC peptides are eluted from immunoaffinity beads, the elution will also, at minimum, elute the MHC molecules as well, which are comprised of HLA alpha chains (∼50 kDa) and β2m (12 kDa). While these contaminants are unavoidable, many more contaminants including phosphatase inhibitors, nucleic acids, lipids, and abundant cellular proteins can also be present in the eluate. Additionally, some level of polymer contamination is inevitable when performing a multistep process. Together, these contaminants can hamper identification of MHC-associated peptides through ion suppression, negative impacts on chromatography, and/or co-isolation of precursor ions. Currently, MHC isolation protocols attempt to remove these contaminants with C18 material for an offline cleanup prior to inline analytical separation. With extensive washing, C18 can remove most of these; however, issues caused by other contaminants such as PEG polymer, NP-40, Triton X-100, etc. are not addressed by C18 columns. We identified this as an area with substantial potential for improvement in immunopeptidomics. The primary C18 method of choice to desalt HLA-associated peptides prior to analysis is STAGE tips (44). As with other reverse phase methods, these STAGE tips associate better with hydrophobic moieties. Therefore, proteins are eluted by increasing the organic concentration of the eluent using 30 to 50% acetonitrile. However, we observed significant losses of hydrophilic peptides using this technique. Thus, we reasoned that a hydrophilic interaction chromatography (HILIC) resin could potentially decontaminate samples while retaining hydrophilic phosphopeptides. Introduced by Alpert in 1990 (45), PHEA (Poly LC) was the first resin used for HILIC, though was replaced by other materials due to its lack of reproducibility and poor peak shape during online separation (46, 47). However, these issues can be circumvented using off-line separation. Thus, we designed a spin-tip HILIC cleanup method using PHEA beads packed on a glass fiber filter. Unlike current cleanup methods in the field, this material can remove polymers and detergents from the sample, allowing better ionization and analysis of antigenic peptides.

**Fig. 3:**
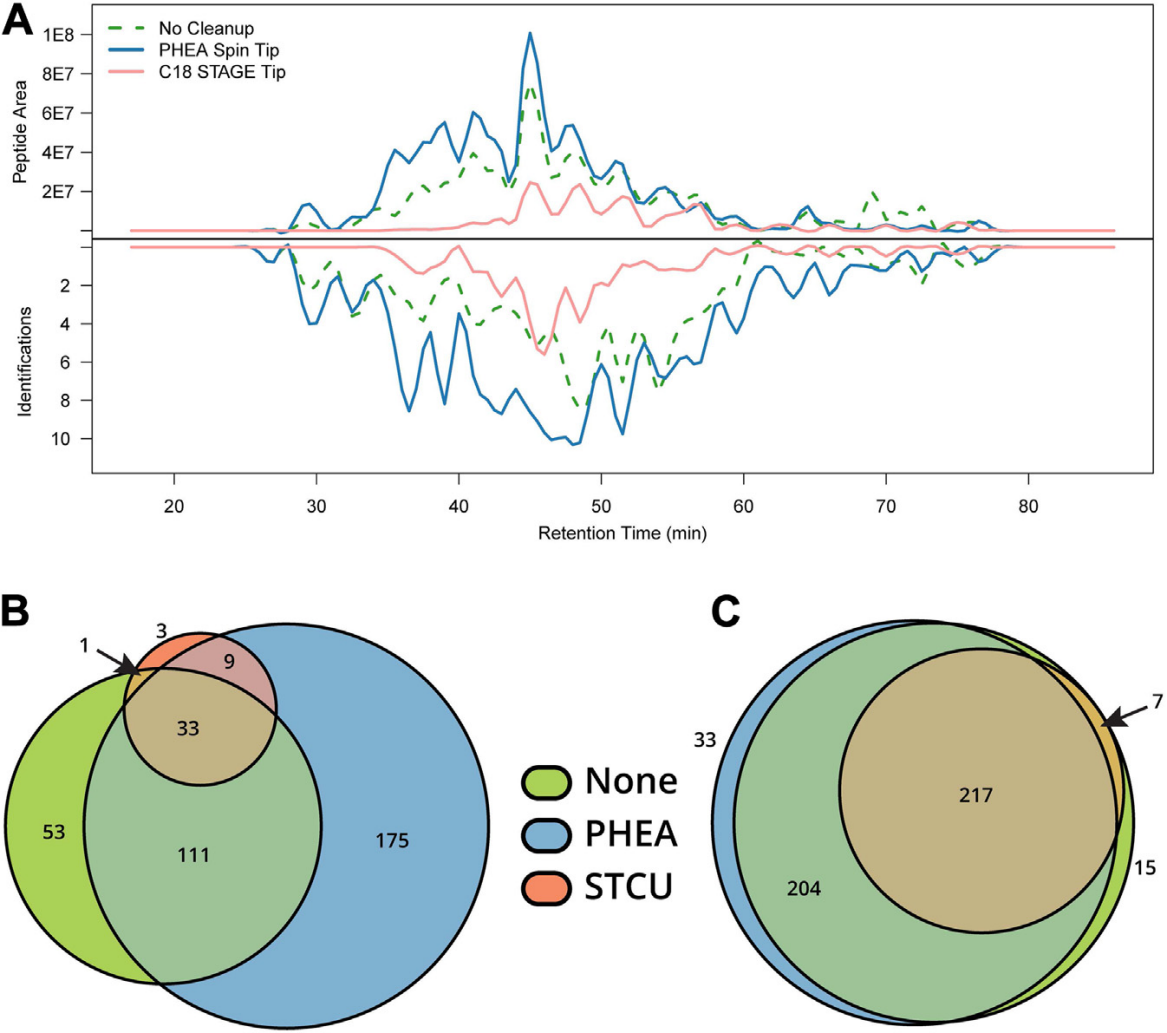
**Comparison of cleanup techniques for a heavily contaminated sample containing HLA peptides from cell line VMM39.** HLA-associated peptides were isolated from VMM39 cells, followed by cleanup with C18 or PHEA STAGE tips. Samples were analyzed were performed on an LTQ FT Ultra. Data were searched using the Byonic node of Proteome Discoverer 3.3 against the human proteome. Identifications were considered confident if they had a Byonic score greater than 300, |logProb| greater than 3, and Proteome Discoverer considered the identification to be high confidence. Peptide quantification and chromatographic alignment were performed by Proteome Discoverer. *A*, PHEA cleanup (*blue trace*) allows for a higher peptide area and greater number of peptide identifications than no cleanup (*green trace*) or C18 (*pink*). *B*, Euler plot demonstrating the overlap in confident identifications between the three techniques. *C*, Euler plot showing overlap when the peptides were found by mass and retention time but were not identified. HLA, human leukocyte antigen; PHEA, polyhydroxyethyl aspartamide; STAGE, stop and go extraction.

To benchmark the PHEA cleanup against traditionally used C18 STAGE tips, we employed a melanoma cell line (VMM39) that was heavily contaminated with polymer and detergent peaks. As seen in Figure 3, PHEA allowed for noticeably more identifications than either the control (*i.e.*, no treatment) or C18, particularly in the portions with polymer contamination. For this sample, PHEA allowed a 130% recovery of total peptide signal, while C18 had 41%. While legitimate recoveries over 100% are not possible, it is a frequent occurrence after PHEA cleanup, likely due to removal of suppressive elements. Thus, the apparent recovery is over 100%, but the true recovery is unknown. The PHEA cleanup also allowed for identification of 402 peptides, compared to 277 identified without a cleanup and 131 detected after a C18 STAGE tip. We repeated this analysis with another cell line bearing much more hydrophobic HLA alleles (*i.e.*, A∗02) and observed similar results (supplemental Fig. S1). We note that these experiments were not performed in triplicate and thus we cannot make statistical claims about these results. That said, in these experiments, we observed that PHEA can be used to decontaminate samples and improve peptide identifications.

**Fig. 4:**
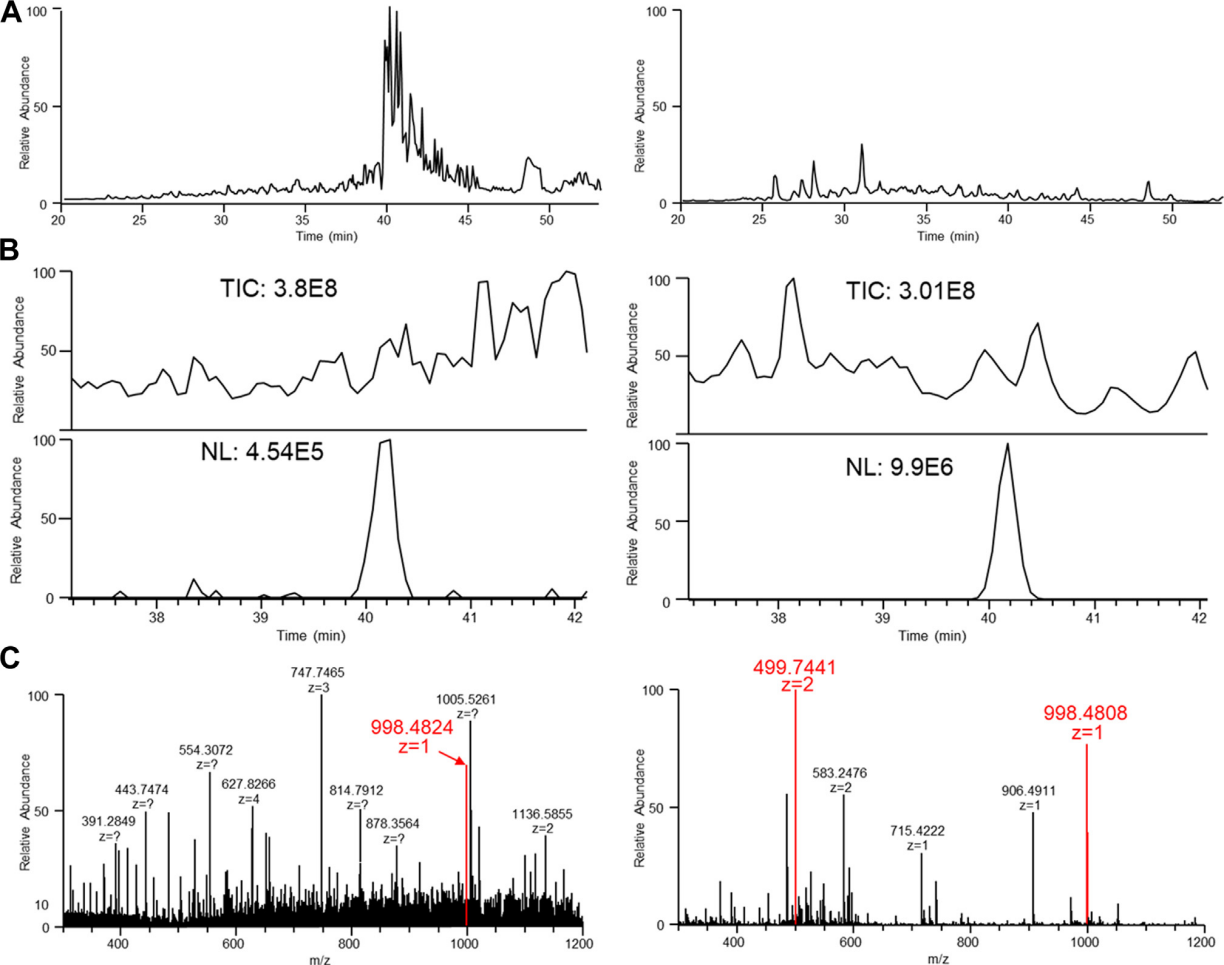
**Ion suppression in a contaminated sample from THP-1 associated HLA peptides is reduced by HILIC cleanup.** Shown here are comparisons of chromatograms and spectra after a STAGE tip cleanup (*left*) and subsequent HILIC cleanup (*right*) for THP-1 cell line. *A*, total ion current for the chromatographic gradient showing the removal of β2m (eluting at ∼40 min prior to HILIC cleanup). *B*, TIC (*top*) and extracted ion chromatogram for peptide SLPDFGISY (*bottom*) showing 20× signal intensity for the peptide after PHEA cleanup due to removal of suppressants. *C*, MS1 spectra at the peptide retention time shows significant noise that is removed after PHEA cleanup. With removal of the coeluting contaminants, the peptide accepted an additional charge, which allowed it to be selected for fragmentation. The peptide was then the most abundant species eluting at the time. β2m, β-2-microglobulin protein; HLA, human leukocyte antigen; HILIC, hydrophilic interaction chromatography; PHEA, polyhydroxyethyl aspartamide; STAGE, stop and go extraction; TIC, total ion chromatogram.

Further, we determined that PHEA could lower ion suppression for low abundance peptides. For instance, a leukemia cell line (THP1) was previously analyzed following a STAGE tip protocol, but only five peptides were confidently identified due to severe contamination. A subsequent cleanup using PHEA on the eluate of this cleanup removed significant contamination, allowing confident identification of 270 unique peptides. Prior to HILIC cleanup, polymers coeluted with the peptides, which both decreased the chance of a peptide being selected for fragmentation and suppressed the signal of the peptide (Fig. 4, *left*). The suppression also caused a decreased overall abundance as well as a lower charge distribution, visible in the MS1 spectrum. This was so substantial without PHEA cleanup that the precursor mass was not selected for fragmentation, whereas afterward, a clear, unambiguous identification was made (Fig. 4, *right*).

**Fig. 5:**
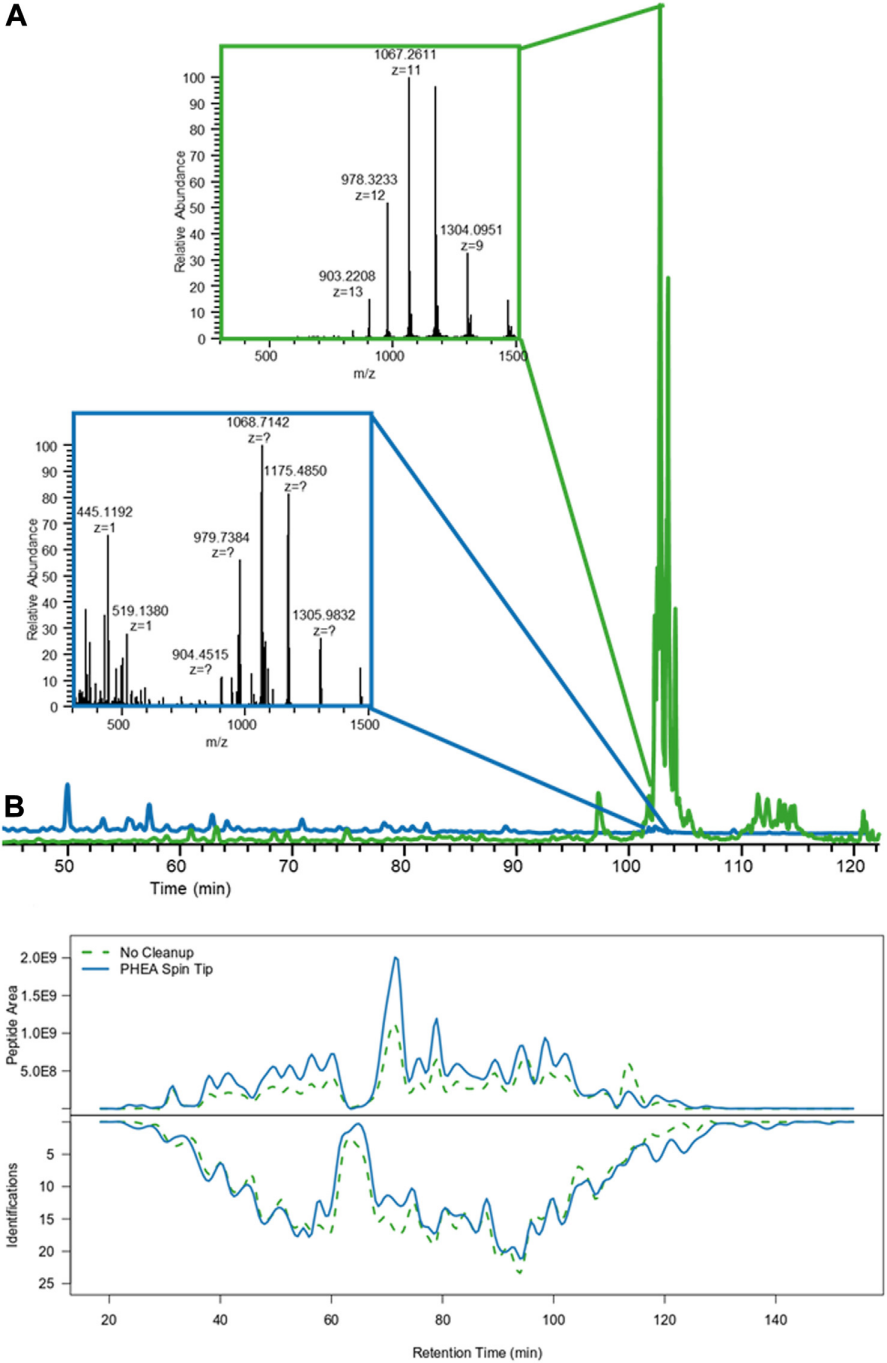
**Selective removal of beta-2-microglobulin from an HLA-associated peptide sample.** Unlike reverse phase removal of β2m from samples, PHEA is able to retain peptides that elute after the protein despite its preference for hydrophilic molecules. *A*, total ion chromatogram (TIC) before (*green*) and after (*blue*) PHEA cleanup of a HeLa sample; after PHEA, the contaminant protein was only present at approximately 1% relative abundance. *B*, without removal of β2m, which elutes at ∼104 min, peptide identifications drop dramatically thereafter (*green*), whereas PHEA continues to have identifications at 140 min (*blue*), indicating selective separation of proteins from peptides. HLA, human leukocyte antigen; PHEA, polyhydroxyethyl aspartamide; TIC, total ion chromatogram.

Finally, most the reversed-phase cleanup protocols attempt to remove intact proteins (*e.g.*, β2m) by eluting from a C18 cleanup with ∼30 to 40% acetonitrile. In our protocol, 50% acetonitrile with 10 mM ammonium formate or 0.1% acetic acid (∼pH 3) was used to elute peptides from the spin tip. One downside of this elution method is that β2m and the HLA alpha chain will elute as well, but another benefit of PHEA is that we can separate these intact proteins from the desired HLA peptides. Here, proteins remain bound to the material and require chaotropic conditions to elute from the column; if interested in the downstream study of these proteins, the tip can then be moved to a new collection tube before eluting with unbuffered 0.2% formic acid. This separation allows for removal of proteins as low as 8 kDa while retaining late-eluting peptides for analysis. As seen in Figure 5*A*, in an HLA-associated peptide enrichment using HeLa cells, β2m eluted at ∼105 min, while peptide identifications continued until ∼140 min after PHEA cleanup. While reversed-phase methods can allow for the removal of β2m and HLA alpha chains, they also result in the removal of all peptides that elute after β2m. In hydrophobic motifs such as A∗02, approximately 15 to 20% of the peptide identifications elute after β2m (Fig. 5*B*), so this method of separation is not ideal (supplemental Fig. S1). One workaround for this is to use molecular weight cut-off filters to remove proteins in a size-dependent manner rather than by hydrophobicity, but those incur significant losses when working with small sample sizes (48). In contrast, the PHEA column still provides this benefit when working with much smaller samples. Overall, we demonstrated that PHEA cleanup is a superior method to a C18 STAGE tips for preparation of samples prior to MS analysis and/or phosphopeptide enrichment for MHC-associated peptides.

### Phosphopeptide Enrichment Resin Selection

Peptides with PTMs are estimated to be present at approximately 0.1 to 1% of the total HLA-associated peptide population (25, 26, 27, 28). Due to their extremely low abundance coupled with the complexity and quantity of unmodified peptides present on the surface of cells, enrichment is necessary to identify any low abundance phosphorylated MHC antigens. The main method for performing enrichment by the Hunt Lab is IMAC (35, 49, 50, 51, 52, 53). Phosphopeptide enrichment with IMAC is performed through conjugation of a chelator such as iminodiacetic acid (IDA) or NTA with a trivalent metal ion, most commonly iron (III) (50). Negatively charged phosphorylation associates with the positively charged metal. However, depending on the pH of the loading solvent, the peptide can carry additional negative charges at acidic residues and the C terminus, leading to significant nonspecific binding. To counteract this, the pH of the loading solvent can be decreased, but this also lowers the affinity of the phosphate group for the metal; thus, our protocols use esterification to circumvent this issue. Fischer esterification adds a methyl group to carboxylic acids in the peptide but does not esterify the phosphate. This allows for the enrichment to be performed at higher pH (∼5), which means that the phosphate will carry two negative charges while the rest of the peptide has 0 negative charges, allowing for a highly specific enrichment.

Elution from Fe-IMAC is most commonly enacted through competition with phosphate buffer. This is undesirable for our purposes, as phosphate is not volatile and requires additional desalting before MS analysis. Therefore, we elute from the Fe-IMAC columns using ascorbic acid, which reduces iron (III) to iron (II), causing it to lose affinity for the phosphate. Ascorbic acid is sufficiently volatile that we can attach a PC to the IMAC column and elute directly onto the column. The sample is then rinsed on the same column used for analysis. This minimizes the steps necessary when the enriched sample is at the highest risk for losses.

IDA was the original material used for metal immobilization for phosphopeptide enrichment (54). However, after its introduction in the late 1980s, NTA quickly became a more popular support due to its higher specificity (55, 56). The higher specificity is achieved because the NTA has an additional ligand arm, which imparts approximately 1.5e5 times the binding affinity for iron (III) (57). The pKa of each ligand arm is approximately 2, below which, affinity decreases dramatically. Therefore, it is impossible to load at a low enough pH to protonate glutamate (pKa = 4.1), aspartate (pKa = 3.9), and the C terminus (pKa∼2) while keeping the ligand fully deprotonated. This causes iron (III) to leach from the column, which decreases phosphopeptide retention. The additional chelating arm on the NTA gives an additional negative charge, which should decrease leaching at low pH. Using esterification, this issue is circumvented, as the acidic regions are esterified rather than protonated. This allows samples to be loaded at a higher pH, which should both decrease leaching in addition to increasing the affinity of the phosphate for iron by providing an additional partial negative charge on the phosphate group.

Previous comparison of the two chelating ligands indicated that they enrich complementary phosphopeptides (50). In that study, an ovarian cancer cell line (FHIOSE) and a colorectal cancer patient sample (CRCLM03T) were compared using an LTQ-Orbitrap instrument. However, these comparisons only considered the results from search algorithms (*i.e.*, identifications from fragmentation spectra), as opposed to investigating the presence of the intact mass in the MS1. While it remains a useful instrument, the LTQ-Orbitrap is not able to collect MS2 scans as quickly as the newer Orbitrap Fusion Tribrid instruments. This makes it much more common for a peptide to be present in a full mass spectrum but not be selected for fragmentation by the mass spectrometer. Given these instrument improvements, we reasoned that revisiting the complementarity of the two techniques was warranted. Thus, we compared a colorectal carcinoma cell line grown in a mouse (colo205m) and two hepatocellular carcinoma clinical samples (LL5176T, LL4857T).

The enrichment using NTA allowed for more identifications than IDA in all cases (supplemental Fig. S2). The major difference between the two resins was in the identification of peptides present at an abundance of less than 1 fmol. Between the three samples, 55% were identified using both resins, 41% only by NTA and 4% only by IDA. The majority of peptides identified only in IDA enrichments were low-level peptides that were present at a similar or higher abundance in the NTA chromatograms and did not have assigned charge states, eliminating them from selection for fragmentation. Only one peptide identified solely using IDA was not present in the corresponding NTA enrichment by mass and retention time (supplemental Fig. S3). Therefore, based on this analysis, NTA resin afforded superior enrichment over IDA, and we recommend its use for HLA-associated phosphopeptide enrichment. That said, we note that these experiments were not performed in triplicate and thus we cannot make any claims about statistical significance.

### Optimizing NTA-Based Enrichment

As described in the previous section, we determined that NTA is a superior resin. Despite this fact, the Hunt lab primarily returned to using IDA because the fused silica microcolumn IMAC protocols caused significant issues with column clogging. We reasoned that the benefits provided by the NTA protocol warranted investigating the problem, especially during the sample preparation process. We hypothesized that the column blockages were caused by DNA nonspecifically bound to the NHS beads during immunopurification. Thus, after identifying a sample that caused irreversible column blockage, we tested this theory by first drying the sample to remove the organic loading solvent. We then resuspended the sample in Tris buffer with 2 mM MgCl_2_ for treatment with Benzonase nuclease, which digests both RNA and DNA into small 1 to 5 base pair fragments. The addition of MgCl_2_ is the key, since EDTA is often present in the lysis buffer. After this digestion, the sample was desalted to remove nonvolatile Tris and MgCl_2_ as well as most of the small, hydrophilic nucleic acid fragments. The subsequent enrichment was straightforward. Interestingly, in the MS analysis, we noticed proteins eluting toward the end of the gradient (supplemental Fig. S4). We were able to identify these proteins as fragments from histone H2B and H4, further confirming the hypothesis.

Phosphopeptide standards were recovered at 39% and 34 MHC-associated phosphopeptides were identified. The presence of contaminant proteins (*e.g.*, histones) interfered with peptide identification, so we determined that it is best to perform Benzonase treatment during cell lysis so the contaminant proteins will be removed prior to the immunopurification. In following immunopurifications, we found that samples lysed and immunopurified in a Tris buffer including 100 U/ml Benzonase nuclease and 2 mM MgCl_2_ alleviated any problems with clogging, thus allowing NTA to be used more effortlessly. Additionally, fewer contaminant peptides were observed in the initial sample screening. Taken together, we recommend the addition of Benzonase nuclease in immunopeptidomic sample preparations to improve IMAC enrichment and downstream MS analysis.

### Decreased Length of Enrichment Procedure

In theory, the previous IMAC protocol took 5 h to complete, followed by LC-MS/MS analysis (2 h), thus required at least 7 h. However, as discussed above, issues often arose when preparing the IMAC column, including sample loading, column packing, etc. putting the upper limit of the experiment close to 12 h. Thus, we sought to shorten the length of time necessary for phosphopeptide enrichment by optimizing the time for Fischer esterification and iron activation steps. In total, we reduced the overall bench time by 50% and greatly improved throughput of our IMAC protocol.

Prior to IMAC, our protocol requires a Fischer esterification to neutralize carboxylic acids in peptides, thus making the enrichment more selective for phosphorylated peptides. We previously performed this reaction by adding anhydrous 3M HCl in methanol, reacting for 1 h, drying (∼1 h), and repeating. However, Fischer esterification is a fast reaction, especially when the acid and alcohol are present in such excess. Thus, we found that reacting for 15 min instead of 1 h reaches the same level of derivatization (∼99%) and shortens the drying step to ∼20 min. This is likely due to the generation of water as a side product of the reaction, as it is less volatile and takes longer to evaporate than the methanolic HCl. The removal of water more rapidly also reduced the generation of side reactions, such as deamidation of asparagine and glutamine, as well as the possibility of reversing reactions. This reduced our esterification protocol from 4 h to approximately 1.5 h without sacrificing completeness.

Using the previous protocol, IMAC resin was activated on the same day as the enrichment, but NTA resin can be activated before the enrichment and appears to retain its activity for at least 1 month. This reduced the amount of time needed overall, as the activated resin can be used for multiple enrichments. Further, due to the stronger affinity of iron for NTA, we theorized that the resin would retain iron even with an increased flow rate. Previously, most steps were carried out at a flow rate of 0.5 μlmin^−1^. Here, we increased the flow rate prior to sample loading to 2 μlmin^−1^ and after sample loading to 1 μLmin^-1^. This change to the protocol showed no noticeable changes in recovery of phosphopeptide standards compared to the previous protocol, while decreasing time at the bench substantially. Between the increased flow rates and bulk activation prior to the day of enrichment, this protocol can be completed in approximately 2.5 h, followed by a 2-h LC-MS/MS analysis. Since the Benzonase treatment prevents most sample-related clogs, sample loading times are more predictable and flow rates remain constant. Overall, these improvements have led to more straightforward, fast, and reproducible phosphopeptide enrichment.

### Total List of Post-translationally Modified MHC-Associated Peptides

To date, we identified 2201 MHC-associated phosphopeptides (supplemental Table S1) across 180 samples and 18 tissue types (supplemental Table S5); cancer type abbreviations can also be found in supplemental Table S6. supplemental Table S1 details the HLA peptide sequences, any abnormalities in the identified sequence (compared to UniProt sequences), the position of the modification, and any other modifications beyond phosphorylation that were detected. This included 2099 site-localized monophosphorylated peptides, 30 site-localized diphosphorylated peptides, and 72 partially localized or ambiguous phosphopeptides. We observed 1633 individual phosphosites, including 329 that are not currently reported by UniProt, PhosphoSitePlus, or the PRIDE phosphoproteome map. For posterity we also included the UniProt number of the originating protein and the phosphosite position on the protein. When possible, we assigned a potential HLA allele; in some cases, the peptide could be assigned to more than one and thus the column is left blank.

Beyond phosphorylation, many other PTMs are known to be dysregulated in cancer. For instance, O-GlcNAc is a dynamic PTM that operates similarly to phosphorylation, often modifying the same sites on a protein in different cellular conditions. O-GlcNAc dysregulation in cancer is often linked to increased activity of the hexosamine biosynthetic pathway, enhancing protein O-GlcNAcylation, which promotes tumor growth, metastasis, and therapy resistance. Aberrant GlcNAc modification of key signaling and transcriptional proteins alters cellular processes such as proliferation, apoptosis, and metabolism, contributing to cancer progression (58). As with phosphorylation, where we were the first to identify a phosphopeptide and neoantigen (59), we were among the first to identify O-GlcNAcylated HLA-associated peptides (36, 60) and, in total, detected 116 glycopeptides (77 site-localized; supplemental Table S2). Some of these antigens were able to stimulate immune responses in healthy individuals, suggesting their potential as cancer neoepitopes for immunotherapy (36). Interestingly, many of these glycopeptides were also methylated. Protein methylation is frequently dysregulated in cancer due to mutations or altered expression of methyltransferases and demethylases, leading to aberrant methylation patterns on histones and non-histone proteins. These changes disrupt gene expression, DNA repair, and signal transduction, driving tumorigenesis, metastasis, and resistance to therapies (61). In combination with peptides that bore only methylation, we identified 88 HLA-associated peptides (supplemental Table S3). Finally, kynurenine is a PTM often dysregulated in cancer due to overactivation of the tryptophan–kynurenine pathway, driven by increased expression of enzymes like IDO1, TDO, and KYN2. Elevated kynurenine levels suppress antitumor immune responses by activating regulatory T cells and aryl hydrocarbon receptor signaling, fostering an immunosuppressive tumor microenvironment (62, 63). We detected 58 site-localized kynurenine-containing peptides (supplemental Table S4). To our knowledge, these represent the first examples of this modification on HLA-associated peptides, and most of the PTM sites are not currently reported in UniProt. Taken together, the Hunt laboratory has established robust protocols to identify PTM-bearing peptides associated with HLA molecules. We again hope these lists can serve as useful tools in identifying targets for potential cancer immunotherapies.

### Bioinformatics Analysis of MHC-Associated Phosphopeptides

**Fig. 6:**
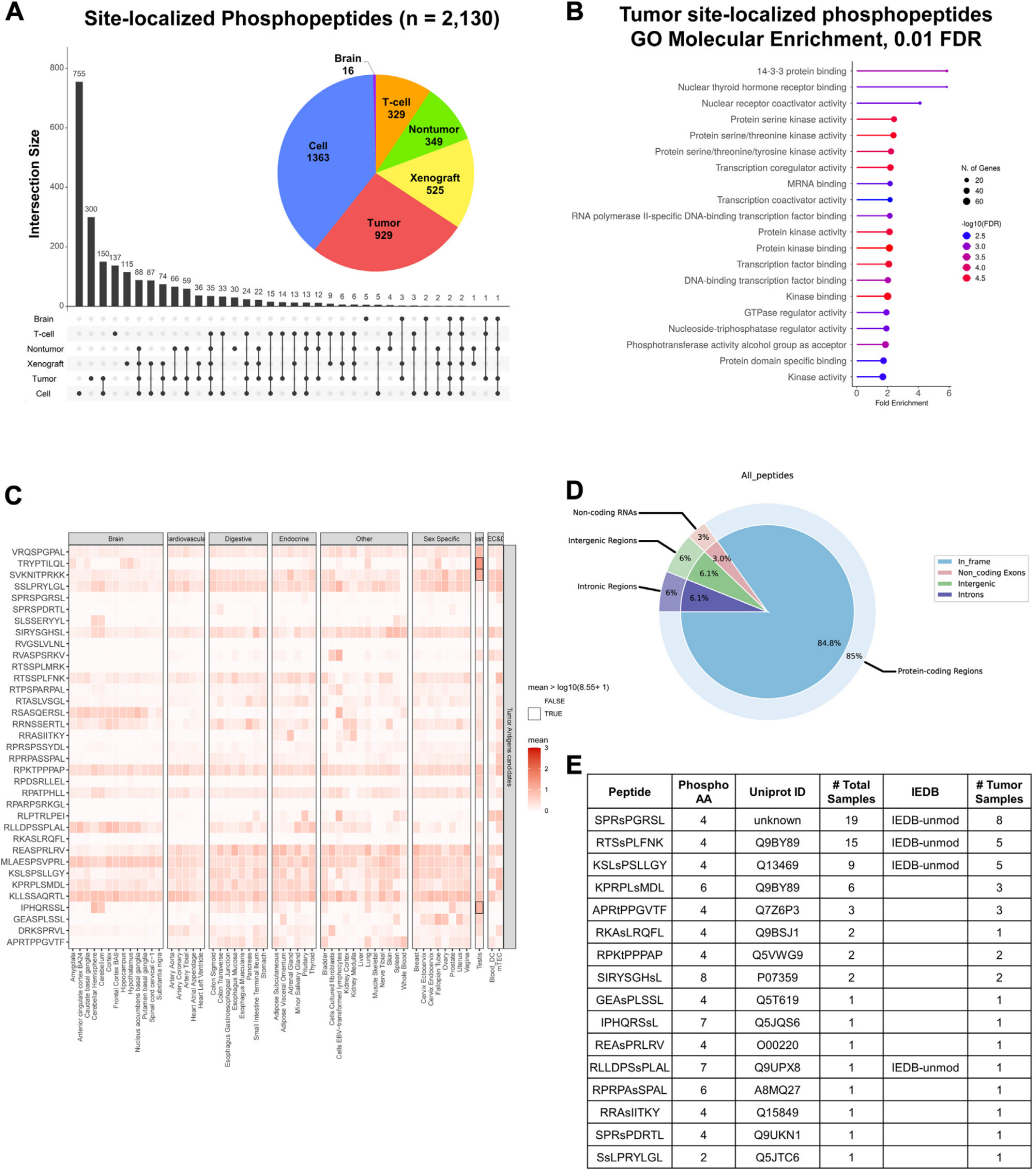
**Tumor-associated phosphopeptides nominated for immunotherapeutic study.***A*, site-localized MHC class phosphopeptides were categorized into six sample groups. Patient tumor tissue constituted ∼50% of the samples tested and identified the second greatest number of phosphopeptides. Immortalized human cell lines identified the most phosphopeptides, however many of these were not observed in other sample groups. Xenograft cell lines, nontumor patient tissue, and T cells each identified several hundred phosphopeptides, while cadaverous brain tissue identified less than 20. *B*, tumor-associated phosphopeptides were found to be enriched in proteins involved in kinase signaling and transcriptional regulation when compared to MHC Class I peptides from the HLA Ligand Atlas. *C*, the likelihood of HLA presentation was estimated using BamQuery, which identified that 33 phosphopeptides may originate from proteins with low expression across healthy human tissues. *D*, of the 33 phosphopeptides with low HLA presentation likelihood, 28 peptides aligned to protein-coding genomic regions while five were predicted to come from intronic, intergenic, and noncoding regions. *E*, we nominate 16 tumor-associated phosphopeptides as candidate tumor antigens, including eight that have been observed in more than one patient tumor and four that were previously studied for immune reactivity as unmodified antigens. HLA, human leukocyte antigen.

Out of the 2130 site-localized phosphopeptides, we identified the greatest number of peptides from immortalized human cell lines (n = 1363), followed by patient tumor tissue (n = 929), human cell line mouse xenografts (n = 525), nontumor patient tissue (n = 349), T cells (n = 329), and cadaverous brain tissue (n = 16) (Fig. 6*A*). Although cell line–associated peptides comprise the majority of the phosphopeptide data, over half of these peptides were uniquely observed in immortalized cell line samples. By comparison, two-thirds of tumor-associated phosphopeptides were also observed in another sample type. We postulate that phosphopeptides identified from tumor tissue, including those that were observed in other sample groups, may be more representative of the underlying disease than peptides that were identified solely in cancer cell lines, which may be skewed to represent proteins disrupted by cell immortalization or *in vitro* growth conditions.

We performed gene set enrichment analysis to characterize the molecular function of phosphoproteins sampled by the MHC class I complex in patient tumors. Compared to unmodified MHC class I peptides identified by the HLA Ligand Atlas, tumor-associated phosphopeptides are enriched in proteins involved in kinase binding, cell signaling, and transcriptional regulation (Fig. 6*B*).

We next investigated which phosphopeptides may come from source proteins that are lowly expressed in normal tissue using BamQuery. Out of the entire list of MHC-associated phosphopeptides, 35 were predicted to have low expression across healthy genotype-tissue expression tissues, medullary thymic epithelial cells, and dendritic cells (Fig. 6*C*). Roughly 85% of these peptides map to known protein coding regions, although several peptides were predicted to originate from intronic, intergenic, and noncoding RNA regions (Fig. 6*D*). Two peptides, RPARPSRKGL and RTSSPLMRK, were excluded from further analysis due to zero transcript expression across all queried samples. Of the 33 phosphopeptides, 16 were observed in tumor samples, including eight that were found in two or more tumors and four that have been previously studied for immune reactivity as unmodified peptides (Fig. 6*E*), suggesting this subset of phosphopeptides may be the best candidates for future immunogenicity investigations.

## Discussion

Here, we describe optimized protocols for the enrichment and identification of PTM MHC class I–associated peptides, with a focus on phosphopeptides. The identification of tumor-associated MHC peptides, including those with PTMs, are of high interest as possible biomarkers or immunotherapeutic targets since cancer and other chronic diseases can evade detection by the immune system, in part, by dysregulation of cellular signaling. Additionally, it is possible that proteins containing PTMs may undergo antigen processing differently. Therefore, the identification of disease associated with MHC-associated PTM peptides will further our understanding of the role that these peptides play in disease progression and possible treatment.

Currently, the identification of MHC-presented phosphopeptides is often done without biochemical enrichment using bioinformatic approaches (64, 65, 66, 67). Although these approaches yield identifications, they may be biased toward only the highest abundant phosphopeptides, and often only obtain 10s to 100s of phosphopeptides per sample. It is also noteworthy that many of these datasets are generated without phosphatase inhibitors, which may also bias the observed MHC phosphopeptides. Therefore, our improvements to the phosphopeptide enrichment protocol for MHC peptides have enabled more comprehensive coverage and understanding of MHC-presented phosphopeptides. Previously published MHC phosphopeptide enrichment protocols were lengthy and difficult to scale (50), leading us to investigate a number of aspects of sample preparation and processing. For instance, by changing the metal chelating ligand, we increased flow rates during enrichment, which decreased the enrichment time by half. Additionally, treatment of samples with nuclease prior to immunoprecipitation decreased contaminants present in the sample, which prevented clogging during enrichment. This facilitated faster and more consistent sample loading time as well as reducing contamination from DNA and DNA-binding proteins. Finally, we describe a hydrophilic interaction–based method for sample cleanup that enabled improved retention of hydrophilic peptides, removal of intact proteins, and removed polymeric/detergent contaminants. Overall, these optimizations have allowed for improved identification of unmodified MHC peptides as well as PTM peptides, particularly for tissues with low MHC expression and/or less starting material.

The identification of an MHC-associated phosphopeptide on a tumor sample does not directly indicate that it is likely to be immunogenic and not expressed by other healthy cells in the body. Therefore, additional evaluation of tumor-associated MHC phosphopeptides is required to nominate peptides that are most likely to be potential immunotherapeutic targets. One commonly performed immunogenicity evaluation is done by using the ELIspot assay. In short, either patient or healthy donor peripheral blood mononuclear cells (PBMCs) are exposed to synthetic peptide antigens in microplates precoated with anti-IFN-γ antibody. After incubation to allow for cytotoxic T-lymphocytes in the PBMCs to recognize the antigen and proliferate, cells are restimulated with synthetic antigen before reaction with a detection reagent. Based on IFN-γ production compared with positive and negative controls, it is possible to determine if the patient or healthy donor has CD8+ T-cell responses against the assayed peptides (36, 52). Another widely used method to test for immunogenicity of MHC-associated peptides that can be applied to phosphopeptides is tetramer-based assays, which can also be used on either healthy donor or patient stimulated PBMCs (68, 69, 70). Although there are other approaches to identify CD8+ T-cell targets, such as T-scan (71), assays relying on genomically encoded MHC peptides may miss post-translationally modified MHC peptides. Regardless of the assay used to evaluate MHC phosphopeptide immunogenicity, additional evaluation of source protein expression and PTM status in other cell types in the body is required to nominate an MHC phosphopeptide as a potential immunotherapeutic target.

It remains difficult to nominate MHC phosphopeptides as putative immunotherapeutic targets, even if they are shown to be immunogenic, because there is not a well-established “normal” MHC phosphopeptide atlas or reference database (72, 73), as there is with unmodified MHC peptides (41). Although many phosphoproteomic datasets have been produced with tryptic digestion, most are generated from tumor tissue. Even those that have paired adjacent normal tissue do not represent all possible phosphoproteins expressed within the body, especially given the diverse cell types that exist. Additionally, since phosphorylation is rapidly turned over, it is possible that phosphoproteomics datasets might not capture phosphosites sampled by the MHC class I pathway. Further, the peptide binding grooves of MHC class I molecules dictate which peptides can be presented, which are often different than those generated by tryptic digestion. Because of these challenges, we attempted to nominate MHC phosphopeptides that were likely to be lowly expressed on other tissue types using their unmodified source proteins as an input for BamQuery (42). We hypothesized that if the unmodified source protein had low expression in benign tissues (72, 73), and then the post-translationally modified form is likely to have similar behavior. After filtering all identified phosphopeptides using BamQuery, we then chose a subset (n = 16; Fig. 6) that appeared in multiple tumor samples and tumor types, as these may represent shared antigen targets that are desirable for immunotherapeutic approaches. We acknowledge that additional immunogenicity follow-up is required to further support these peptides as putative immunotherapeutic targets, and our goal is to present this list of modified peptides to the immunopeptidomics and immunology communities in hopes that others will take advantage of this resource in the development of future immunotherapies.

Overall, the Hunt laboratory has revolutionized the field of immunopeptidomics by applying advanced mass spectrometry techniques to uncover the complexities of antigen presentation, with a particular focus on phosphopeptides and other PTMs. Our work has illuminated which of these modified (and often dysregulated) peptides are presented by MHC molecules, revealing previously unrecognized mechanisms by which the immune system can recognize abnormal cellular processes. By mapping these PTMs, the Hunt lab has provided a blueprint for identifying tumor-specific antigens, which will hopefully be useful for other immunopeptidomic studies. These contributions have paved the way for developing novel therapeutic strategies that harness the immune system to selectively attack cancer cells while sparing healthy tissues. We envision our detailed HLA peptide lists will enable others to do just that.

## Data Availability

We are unable to upload all raw files and annotated spectra given that these data have been collected over the course of 30 years. To demonstrate representative data, we uploaded 11 raw files and the associated search results to PRIDE (PXD061414), along with 70 example annotated spectra. Additionally, we are happy to provide data files upon request.

## Supplemental data

This article contains supplemental data.

## Conflict of interest

The authors declare no competing interests.

## Supplementary Data

SI_Table_1

SI_Table_2

SI_Table_3

SI_Table_4

SI_Table_5

SI_Table_6

SI_Table_7

SI_Figures S1-S4 and Captions of Tables S1-S6
