# Supplementary material for: Identification of Post-translationally Modified MHC Class I–Associated Peptides as Potential Cancer Immunotherapeutic Targets
Source: Mol Cell Proteomics. 2025 Apr 14;24(8):100971. doi: 10.1016/j.mcpro.2025.100971 (PMC12362676; doi:10.1016/j.mcpro.2025.100971)
Supplement: SI_Table_7 [file mmc7.docx]

**Hunt Lab Publications on Immunopeptidomics**

Phosphorylated Peptides are Naturally Processed and Presented by MHC Class I Molecules In Vivo, A.L. Zarling, S.B. Ficarro, F.M. White, J.. Shabanowitz, D.F. Hunt, V.E. Engelhard, J. Exp. Med., 2000, 192, 1755-1762.

Identification of Class I MHC-Associated Phosphopeptides as Targets for Cancer Immunotherapy, Zarling, AL, Polefrone, JM, Evans, AE, Mikesh, LM, Shabanowitz, J, Lewis, ST, Engelhard, VH, Hunt, DF, Proc. Natl. Acad. Sci. USA, 2006,103, 14889-14894.

Phosphorylation-Dependent Interaction Between Antigenic Peptides and MHC Class I:  A Molecular Basis for the Presentation of Transformed Self, Mohammed F, Cobbold M, Zarling AL, Salim M, Barrett-Wilt GA, Shabanowitz J, Hunt DF, Engelhard VE, Willcox BE, Nat. Immunol.**2008** Nov;9(11):1236-43.

Identification of Tumor-Associated, MHC Class II Restricted Phosphopeptides as Targets for Immunotherapy, Depontieu FR, Qian J, Zarling AL, McMiller TL, Salay TM, Norris A, English AM, Shabanowitz J, Engelhard VH, Hunt SF, Topalian SL, Proc Natl Acad Sci USA. 2009 Jul 21;106(29):12073-8. PMCID: PMC2715484

Structural basis for the presentation of tumor-associated MHC class II-restricted phosphopeptides to CD4+ T cells. Li Y, Depontieu FR, Sidney J, Salay TM, Engelhard VH, Hunt DF, Sette A, Topalian SL, Mariuzza RA. J Mol Biol. **2010** June 18;399(4):596-603

MHC class-I associated phosphopeptides are the targets of memory-like immunity in leukemia. Cobbold M, De La Pena H, Norris A, Polefrone JM, Qian J, English AM, Cummings KL, Penny S, Turner JE, Cottine J, Abelin JG, Malaker SA, Zarling AL, Huang HW, Goodyear O, Freeman SD, Shabanowitz J, Pratt G, Craddock C, Williams ME, Hunt DF, Engelhard VH. Sci Transl Med. **2013**Sep 18;5(203):203ra125.

Complementary IMAC enrichment methods for HLA-associated phosphopeptide identification by mass spectrometry, Abelin JG, Trantham PD, Penny SA, Patterson AM, Ward ST, Hildebrand WH, Cobbold M, Bai DL, Shabanowitz  J, Hunt DF, Nature Protoc. **2015** Sep;10(9):1308-18.

The antigenic identity of human class I MHC phosphopeptides is critically dependent upon phosphorylation status. Mohammed F, Stones DH, Zarling AL, Willcox CR, Shabanowitz J, Cummings KL, Hunt DF, Cobbold M, Engelhard VH, Willcox BE. Oncotarget, **2017** Apr 8, (33):54160-54172.

MHC-restricted phosphopeptide antigen: preclinical validation and first in-humans clinical trial in participants with high-risk melanoma. Engelhard VH, Obeng RC, Cummings KL, Petroni GR, Ambakhutwala AL, Chianese-Bullock KA, Smith KT, Lulu A, Varhegyi N, Smolkin ME, Myers P, Mahoney KE, Shabanowitz J, Buettner N, Hall EH, Haden K, Cobbold M, Hunt DF, Weiss G, Gaughan E, Slingluff CL Jr. J Immunother Cancer. **2020** May;8(1):e0000262.

MHC Phosphopeptides: Promising targets for immunotherapy of cancer and other chronic diseases. Mahoney KE, Shabanowitz J, Hunt DF. Mol Cell Proteomics **2021**, Jun 12;20:100112. doi: 10.1016/j.mcpro.2021.100112.

Tumor infiltrating lymphocytes target HLA-I phosphopeptides derived from cancer signaling in colorectal cancer. Penny SA, Abelin JG, Malaker SA, Myers PT, Saeed AZ, Steadman LG, Bai DL, Ward ST, Shabanowitz J,Hunt DF, Cobbold M. Front Immunol. **2021**, Aug 24;12:723566. doi: 10.3389/fimmu.2021.723566.

**Hunt Lab Patents on Immunotherapy of Cancer**

1) Phosphopeptide Antigens Associated with MHC Molecules    US2005/0277161 A1 (Rejected) UVa gave up.

2)  Class I MHC Phosphopeptides for Melanoma Cancer Immunotherapy and Diagnosis PCT/US2011/037699 and WO2011/14 UVA gave up and returned intellectual property to DFH who then patented it.  Owned by PhosImmune and now by Agenus Inc

3)  Phosphopeptides as Melanoma Vaccines, PCT/US2010/033530 and WO2010/129537 A1, Jointly owned by Johns Hopkins U and UVA

4)  Target Peptides for Immunotherapy and Diagnosis PCT/2013/058255 Owned by UVA and licensed to PhosImmune and now Agenus Inc.

5) Identification of MHC Class I Phospho-Peptide Antigens from Breast Cancer Utilizing SHLA Technology and Complementary Enrichment Strategies,  PCT/US2013/042908, US Patent 10,640,535,  2020  Owned jointly by UVA and Oklahoma State University.  Hunt, DF, Norris A, English AM, Hildebrand WH, and Hawkins OE,

6)  Target Peptides for Ovarian Cancer Therapy and Diagnostics, PCT/US2013/075073.  Owned jointly by UVA and Oklahoma State University.

7)  Target Peptides for Colorectal Cancer Therapy and Diagnostics, PCT/US2013/058255 and WO2014/039675 A2. Donald F Hunt, Jeffrey Shabanowitz, Jennifer G Abelin, Mark Cobbold and Sara Amy Penny. Owned by UVA and licensed to Agenus Inc.

8) Target Peptides for (Hepatocellular) Cancer Therapy and Diagnostics. WO 2017/192969, PCT/US 2017/031266 A May 5, 2017. Owned by UVA and Licensed to Agenus.

9)  Identification of Class I MHC Associated Glycopeptides as Targets for Cancer Immunotherapy, US Patent Application 62/202,359 August 2015, Owned by UVA and licensed to Agenus.

10) Compositions and Methods for Treating Diseases and Disorders Associated with Aberrant Regulation of Proteins, Provisional 62/645,221 March 2018. Owned by UVA.
