# Supplementary material for: Identification of Post-translationally Modified MHC Class I–Associated Peptides as Potential Cancer Immunotherapeutic Targets
Source: Mol Cell Proteomics. 2025 Apr 14;24(8):100971. doi: 10.1016/j.mcpro.2025.100971 (PMC12362676; doi:10.1016/j.mcpro.2025.100971)
Supplement: SI_Figures S1-S4 and Captions of Tables S1-S6 [file mmc8.pdf]

Supplemental information for: Identification of post-translationally modified MHC class I-associated peptides as potential cancer immunotherapeutics

Keira E. Mahoney[1,2], Larry Reser[2], Maria Virginia Ruiz Cuevas[3], Jennifer G. Abelin[2,3], Jeffrey Shabanowitz[2], Donald F. Hunt[2*], Stacy A. Malaker[1,2*]

[1] Department of Chemistry, Yale University, New Haven, CT, USA
[2] Department of Chemistry, University of Virginia, Charlottesville, VA, USA
[3] Broad Institute of MIT and Harvard University, Cambridge, MA, USA
* To whom correspondence should be addressed: Stacy A. Malaker (stacy.malaker@yale.edu), Donald F. Hunt (dfh@virginia.edu)

Running title: MHC-associated PTM-peptides for cancer immunotherapy

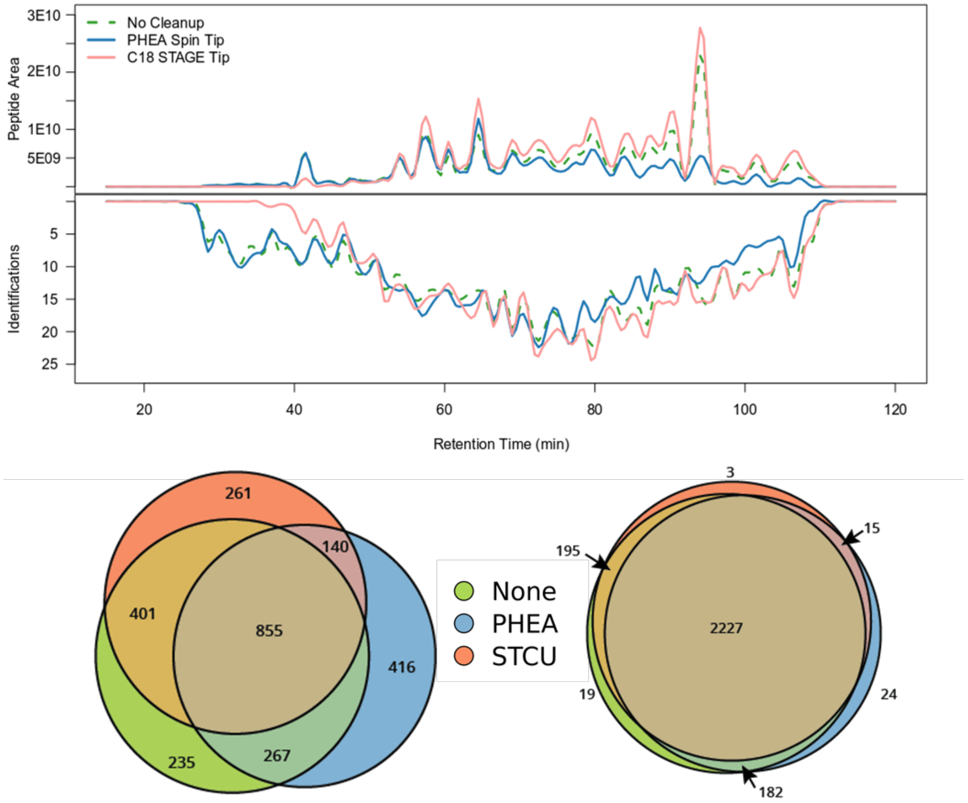

Figure S1. Comparison of cleanup techniques for a hydrophobic HLA type. The top panel includes a rolling average of the chromatographic area assignable to MHC I peptides (top), as well as the number of identifications per minute (bottom) for the sample JYA2. The left Euler plot shows the overlap in confident identifications between the three techniques, while the right shows the overlap when the peptides were found by mass and retention time but not identified. Green is used to show identifications without a cleanup, red for STAGE tip cleanups (STCU), and blue for PHEA spin tip. Notably, approximately 15-20% of identifications for this sample occurred after 105 mins.

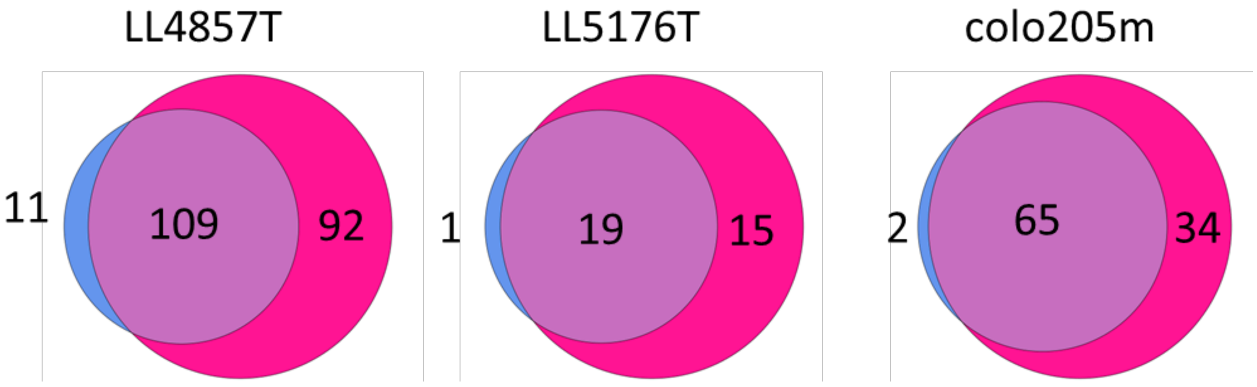

Figure S2. Comparison of MHC phosphopeptide identifications using Fe-NTA or Fe-IDA on an Orbitrap Fusion Tribrid. HLA peptides from 3 cell lines were eluted and enriched using both resin types. Displayed are Euler plots showing overlap of peptides identified by IDA (blue) and NTA (pink) for three MHC class I phosphopeptide enrichments analyzed using an Orbitrap Fusion Tribrid mass spectrometer. In all cases, NTA outperformed IDA with respect to the number of peptides identified.

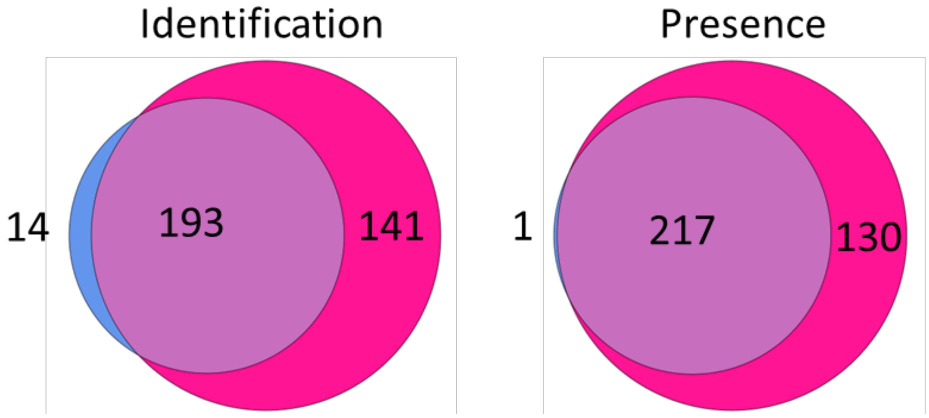

Figure S3. Comparison of identification and presence of peptides between enrichment techniques. Samples were prepared as described in Figure S2. Of the peptides identified only using IDA resin, 13/14 peptides were present in the NTA enrichment by MS1. For those identified only in the NTA enrichment, only 11/141 were present in the IDA enrichment. Thus, we recommend NTA as the resin of choice for HLA phosphopeptide enrichments.

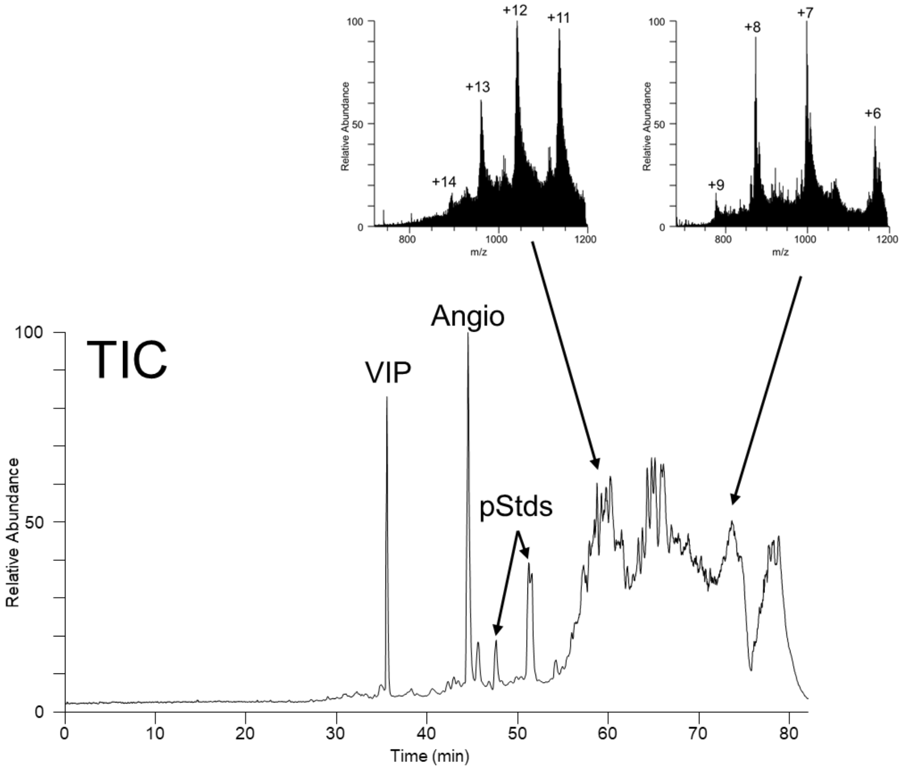

Figure S4. Chromatogram of HEK293Tm after IMAC following Benzonase treatment. HEK293T HLA-associated peptide sample was prepared and then treated with Benzonase, followed by an IMAC enrichment. The chromatogram consisted of standards (VIP, angio) and phosphostandards (pStds) followed by elution of large, modified species. Upon investigation, these species were primarily histones and histone fragments. The TIC (bottom) depicts the summed abundance of all peaks present in each MS1. After elution of standards as peaks between 30-55 minutes, a sharp increase in the TIC was observed. These chromatographic peaks corresponded to proteins and protein fragments from 7-13 kDa, some of which were identified as being histone fragments.

Captions for SI Tables

Table S1. Total list of phosphorylated MHC Class I peptides. Columns 1-4 contain the peptide sequence, comments regarding peptide forms, and the amino acid position and mass addition of each detected modification. Site-localized modifications are separated by semi-colons, while unlocalized sites are indicated within parentheses and separated by vertical bars. Column 5 contains the position(s) of phosphorylated amino acids within the peptide sequence. For peptides that match to a known protein, columns 6-8 contain the Uniprot ID, phosphosite location within the protein sequence, and comments identifying point mutations, deamidations, and unreviewed Uniprot entries. For peptides that do not match a known protein sequence, columns 6-8 remain empty. Columns 9-11 contain sample-related information, including HLA allele information, the sample(s) that the peptide has been identified in, and the type(s) of cancer the peptide has been found in (cancer codes found in Table S6). Column 12 contains a numerical identifier for each peptide.

Table S2. Total list of glycosylated MHC Class I peptides. Columns 1-4 contain the peptide sequence, comments regarding MS/MS evidence for potential sites of modification, and the amino acid position and mass addition of each detected modification. Site-localized modifications are separated by semi-colons, while unlocalized sites are indicated within parentheses and separated by vertical bars. Column 5 contains the position(s) of glycosylated amino acids within the peptide sequence. For peptides that match to a known protein, columns 6 and 7 contain the Uniprot ID and the glycosite location within the protein sequence. For peptides that do not match a known protein sequence, these columns remain empty. Columns 8-10 contain sample-related information, including HLA allele information, the sample(s) that the peptide has been identified in, and the type(s) of cancer the peptide has been found in (cancer codes found in Table S6). Column 11 contains a numerical identifier for each peptide.

Table S3. Total list of methylated MHC Class I peptides. Columns 1-3 contain the peptide sequence and the amino acid position and mass addition of each detected modification. Site-localized modifications are separated by semi-colons. Column 4 contains the position(s) of methylated amino acids within the peptide sequence. For peptides that match to a known protein, columns 5 and 6 contain the Uniprot ID and the methylsite location within the protein sequence. For peptides that do not match a known protein sequence, these columns remain empty. Columns 7-9 contain sample-related information, including HLA allele information, the sample(s) that the peptide has been identified in, and the type(s) of cancer the peptide has been found in (cancer codes found in Table S6). Column 10 contains a numerical identifier for each peptide.

Table S4. Total list of kynurenine-modified MHC Class I peptides. Columns 1-3 contain the peptide sequence and the amino acid position and mass addition of each detected modification. Site-localized modifications are separated by semi-colons. Column 4 contains the position of kynurenine-modification within the peptide sequence. For peptides that match to a known protein, columns 5-7 contain the Uniprot ID, the kynurenine location within the protein sequence, and comments identifying proteins with unreviewed Uniprot entries. For peptides that do not match a known protein sequence, these three columns remain empty. Columns 78-10 contain sample-related information, including whether an HLA allele-specific antibody was used, the sample(s) that the peptide has been identified in, and the type(s) of cancer the peptide has been found in (cancer codes found in Table S6). Column 11 contains a numerical identifier for each peptide.

Table S5. Sample information for MHC Class I peptides. Column 1 contains the sample ID. Columns 2 and 3 contain the MHC Class I alleles expressed by the sample (if known) and the MHC allele-specific antibody used for enrichment. Columns 4 identifies the sample group, while columns 5 and 6 identify the cancer type and tissue source. Columns 7 and 8 indicate when the sample was received and the students who tested it. Columns 9 and 10 flag samples that have gene knock outs or were treated with drug inhibitors.

Table S6. Cancer code information. Column 1 contains the abbreviated cancer code used in SI Tables 1-4. Column 2 contains the associated cancer type used in SI Table 5.
